# One-Step Preparation of Durable Super-Hydrophobic MSR/SiO_2_ Coatings by Suspension Air Spraying

**DOI:** 10.3390/mi9120677

**Published:** 2018-12-19

**Authors:** Zhengyong Huang, Wenjie Xu, Yu Wang, Haohuan Wang, Ruiqi Zhang, Ximing Song, Jian Li

**Affiliations:** 1 State Key Laboratory of Power Transmission Equipment & System Security and New Technology, Chongqing University, Chongqing 400040, China; 18883878345@163.com (W.X.); wangy@cqu.edu.cn (Y.W.); 20134209@cqu.edu.cn (H.W.); zhangruiqi@cqu.edu.cn (R.Z.); songximingchina@gmail.com (X.S.); 2 Postdoctoral Research Station on Chemical Engineering and Technology, Chongqing University, Chongqing 400040, China

**Keywords:** super-hydrophobic, durable, adhesion, corrosive resistance

## Abstract

In this study, we develop a facial one-step approach to prepare durable super-hydrophobic coatings on glass surfaces. The hydrophobic characteristics, corrosive liquid resistance, and mechanical durability of the super-hydrophobic surface are presented. The as-prepared super-hydrophobic surface exhibits a water contact angle (WCA) of 157.2° and contact angle hysteresis of 2.3°. Mico/nano hierarchical structures and elements of silicon and fluorine is observed on super-hydrophobic surfaces. The adhesion strength and hardness of the surface are determined to be 1st level and 4H, respectively. The coating is, thus, capable of maintaining super-hydrophobic state after sand grinding with a load of 200 g and wear distances of 700 mm. The rough surface retained after severe mechanical abrasion observed by atomic force microscope (AFM) microscopically proves the durable origin of the super-hydrophobic coating. Results demonstrate the feasibility of production of the durable super-hydrophobic coating via enhancing its adhesion strength and surface hardness.

## 1. Introduction

Nowadays, with the development of nanotechnology, micro/nano devices have drawn attention to many researchers, and are widely applied in medical, chemistry, biology, electronics, and precise instrument fabricating fields [1,2,3]. However, most of the precise electronic devices are moisture-sensitive, such that water and moisture greatly influences the longevity and the precision. Also, the bioadhesion of the surface, involving different types of interactions between organisms and the surface, make difficulties in further applications in medical and biology [4]. 

In recent decades, it was found that the surface of lotus leaves demonstrated strong hydrophobicity, so-called super-hydrophobicity. The super-hydrophobic surface is defined as the one with a water contact angle (WCA) greater than 150° and water sliding angle (WSA) less than 5°, such that water droplets on it remain spherical and tend to slide off easily [5,6,7]. The reduced area between the droplet and the super-hydrophobic surface leads to a variety of potential applications in anti-fogging [8], humidity-proof [9], bio-surface [10], anti-icing [11], leakage current suppression [12,13], self-cleaning [14], oil/water separation [15], and corrosion protection [16].

In general, the super-hydrophobic surfaces can be achieved by constructing micro/nano-structures on surfaces with low surface energy. Many methods such as chemical etching [17,18], electrochemical deposition [19,20], electrospinning [21,22], phase separation [23,24], plasma treating [25,26], and sol-gel processes [27,28] have been adopted to mimic the “lotus-effect” super-hydrophobic surfaces. The super-hydrophobic surfaces allow a large amount of air entrapment and thus consequently reduce skin friction drag between the surface itself and the droplet. Water droplets tend to roll or slide off the surfaces by tilting or external vibration. The excellent hydrophobicity and self-cleaning properties of super-hydrophobic surfaces are beneficial to reduce water droplets and contamination accumulation, which, therefore, make super-hydrophobic material a prospective candidate to improve anti-pollution performance of outdoor high-voltage insulators in power systems. However, the super-hydrophobic surfaces fail to maintain the super-hydrophobicity because of the fragile micro-structures on them [29,30,31]. Commonly applied inorganic super-hydrophobic coatings like metal oxides [32,33,34] and rare-earth oxides [35] are chemically susceptible. Organic super-hydrophobic coatings [4,36] are short of mechanical robustness, although they have good chemical resistance [37].

Most of the robust super-hydrophobic surfaces are prepared with intrinsically durable media, such as metal meshes and fabrics [38]. The abrasion resistance of a super-hydrophobic surface based on polyurethane elastomer and sacrificial aluminum oxide template was maintained after 10,000 cycles, with a water contact angle (WCA) of above 150° [21]. Furthermore, Alan et al. used a mesh cloth as a reusable template to fabricate a robust super-hydrophobic surface, which remained super-hydrophobic after more than 5500 abrasion cycles at 32.00 kPa [39]. Zhou et al. reported a super-hydrophobic fabric coating made of cross-linked polydimethylsiloxane elastomer, which is capable of withstanding more than 20,000 cycles of abrasion at 12.00 kPa without losing super-hydrophobicity [40]. In addition, Zimmermann et al. prepared durable super-hydrophobic fabrics by a one-step gas phase coating method that maintains the super-hydrophobic property after 1450 cycles of abrasion with the load pressure of 7.8 kPa [41]. Nevertheless, preparing a super-hydrophobic surface with high abrasion resistance without introducing durable media is still challenging.

In this work, we prepared a durable super-hydrophobic coating on glass surfaces by a facial one-step approach and analyzed the hydrophobic properties, mechanical durability, and corrosive liquid resistance. The as-prepared super-hydrophobic surface exhibits a water contact angle (WCA) of 157.2° and contact angle hysteresis of 2.3°. The hierarchical structures on the surface of super-hydrophobic coatings were observed through scanning electron microscopy (SEM) images. The adhesion strength and the hardness of the super-hydrophobic surface were determined to be first level and 4H, respectively. In addition, observed by the atomic force microscope (AFM), the rough structure remains on the super-hydrophobic surface after wearing for 700 mm with a mechanical load of 200 g, which shows that the as-prepared surface maintains the super-hydrophobic ability for long periods. The results demonstrate the feasibility of production of durable super-hydrophobic coatings via enhancing their surface hardness and adhesion strength between the coating and the substrates.

## 2. Materials and Methods

The super-hydrophobic coating in this study was fabricated by incorporation of SiO_2_ nanoparticles with low surface energy into the methyl silicone resin. The SiO_2_ nanoparticles were obtained from the gelation of the nano-silica sol, formed by the composites of the tetraethyl orthosilicate (TEOS) and ammonium hydroxide. Firstly, 30 mL of TEOS dissolved in 350 mL of alcohol was stirred for 24 hours. Then, 18 mL of ammonium hydroxide (volume ratio of distilled water to ammonia was 2:7) was added for the sol generation. Nano-silica aerogel was obtained by drying the nano-silica sol in vacuum at 120 °C for 12 hours and purification with high-speed centrifugation. The chemical reaction is shown below:

(1) $${\left(C_{2}H_{5}O\right)}_{4}\mathrm{Si}\overset{{\;\mathrm{NH}}_{4}\mathrm{OH}\;}{\to }{\mathrm{SiO}}_{2}+C_{2}H_{5}\mathrm{OH}$$

To reduce the surface energy of the SiO_2_ nanoparticles, 3 g of SiO_2_ nanoparticles synthesized above was sequentially mixed with 25 mL of n-hexyl alcohol and 1 g of hydroxyl-terminated polydimethylsiloxane (OH-PDMS) by mechanical stirring. After 24 hours of stirring, 0.08 g of dibutyltindilaurate (DBTD) and 10 mL of n-hexyl alcohol were further added. The chemical graft of the OH-PDMS onto the surface of SiO_2_ nanoparticles proceeded after a further 30 minutes of mechanical stirring. The hydrophobic SiO_2_ nanoparticles were obtained after drying for 2 h at 60 °C. The chemical graft of OH-PDMS on the silica surface is shown below:

(2) $$\mathrm{SiOH}+\mathrm{HO}{\left[\mathrm{SiOSi}\right]}_{n}\mathrm{OH}\overset{\;\mathrm{DBTD}\;}{\to }\mathrm{SiO}{\left[\mathrm{SiOSi}\right]}_{n}\mathrm{OSi}+H_{2}O$$

Two grams of methyl silicone resins and 10 g of hydrophobic SiO_2_ nanoparticles were mixed and ultrasonically dispersed at room temperature for 10 min. The methyl silicone resins/SiO_2_ mixtures were sprayed onto the surface of the slide glass by air spraying using an air spray gun with a nozzle diameter of 0.5 mm and spraying pressure of 0.15 MPa. The spraying distance between the glass slide surface and the nozzle tip was kept around 15 cm. The glass slide with methyl silicone resin/SiO_2_ composite coating was dried in an oven at 100 °C for 1 h and, finally, the super-hydrophobic methyl silicone resin/SiO_2_ composite coating was obtained on the surface of the glass slides.

The hydrophobicity, such as the static water contact angle and the water contact angle hysteresis, of the as-prepared super-hydrophobic coating was measured by a contact angle meter (Drop Meter A-20, Maishi, Ningbo, China). The micro-topography of the as-prepared coating was scanned by scanning electron microscopy (TESCAN VEGA, Zeiss, Oberkochen, Germany) and atomic force microscopy (PARK XE7, Park, Suwon, Korea). The root-mean-square roughness of the super-hydrophobic coating after wearing was calculated from the surface topography. The corrosive liquid tolerance of the super-hydrophobic surface was evaluated by hydrophobicity test after immersion in the acidic and basic liquids. The mechanical durability of the super-hydrophobic coating was tested by measuring the water contact angles and observing the microscopic surface topography after sandpaper abrasion with certain mechanical loads and wear distances, similar to the procedure in the ISO 8251-81 [42] standard. The surface adhesion of the super-hydrophobic coating was evaluated by the adhesion test in ASTM D3359 [43], which classifies the adhesion level of the coating according to the integrity of the coating after being scratched by a multi-blade cutter. In addition, the surface hardness of the as-prepared super-hydrophobic coating was determined by scratch testing based on the ASTM D3363 standard [44], which evaluates the surface hardness of the coating by comparing it with that of the given pencils with a certain hardness by scratching the surface of the coating with fixed pressure.

## 3. Results and Discussion

### 3.1. Surface Micro-Structure

**Figure 1:**
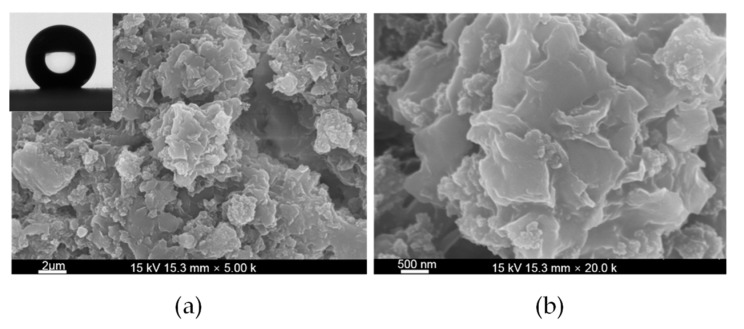
Scanning electron micrograph (SEM) images of the super-hydrophobic methyl silicone resin/SiO_2_ composite surface.

Figure 1a,b show the scanning electron micrograph (SEM) images of the as-prepared super-hydrophobic surface. In Figure 1a, the surface consists of an irregular three-dimensional (3D) micropapilla with a diameter between 2 μm and 4 μm. Furthermore, in Figure 1b, 43 nm to 60 nm nanoparticles are found on the micropapilla. The SEM images further prove that the super-hydrophobic surface has a rough surface.

### 3.2. Surface Wettability

The inset picture in Figure 1 shows the profiles of water droplets on the surface of the methyl silicone resin/SiO_2_ composite coatings. The bare glass substrate exhibited a WCA of 37.5 ± 2°. Compared with the hydrophilic bare glass plate, the water droplets on the surface of methyl silicone resin/SiO_2_ composite coatings are approximately spherical. The static water contact angles on the composite surface are between 154.9° and 160.3°, with an average static water contact angle of 157.2°.

**Figure 2:**
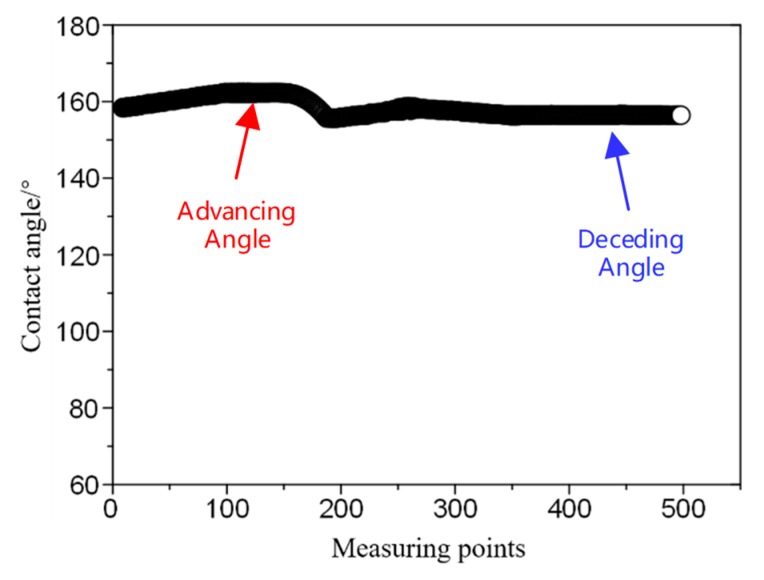
Water contact angle hysteresis of the super-hydrophobic methyl silicone resin/SiO_2_ composite surface.

Figure 2 shows the contact angle hysteresis of the methyl silicone resin/SiO_2_ composite coating determined via one cycle of addition and depression of a water droplet of 10 μL. The contact angle hysteresis curve in the shape of "Z" demonstrates the difference between the advancing and receding water contact angles. As the volume of the water droplet increases, the average advancing water contact angle is measured as 160.8°. When the water droplets stop growing and start to shrink, the receding water contact angle initially increases and then becomes steady, with values between 159.6° and 155.7°, and the average receding water contact angle is 158.5°. The difference between the advancing water contact angle and the receding water contact angle is defined as the water contact angle hysteresis of the methyl silicone resin/SiO_2_ composite coating, with an average value of 2.3°. The low value of the contact angle hysteresis and the great water contact angles of the methyl silicone resin/SiO_2_ composite coating indicates the super-hydrophobic performance of the as-prepared methyl silicone resin surface.

The water droplet stays nearly spherical on the super-hydrophobic surface for the air gaps trapped between the irregular serration of the rough surface and the water droplet. The Cassie Equation [45] was used to quantify the contact area of the super-hydrophobic surface with the air:

(3) $$\cos\theta =f_{1}\left(1+{\cos\theta }_{e}\right)-1$$

where θ is the static water contact angle of the super-hydrophobic coating; f_1_ is the ratio of the solid–liquid contact area of the super-hydrophobic surface; and θ_e_ is the intrinsic contact angle of the methyl silicone resin surface. The static water contact angle of the super-hydrophobic coating is measured as 157.2°. The contact angle of the methyl silicone resin surface is found to be 112°. According to the Cassie Equation, the value of f_1_ can be calculated as 12.49%. Thus, the ratio of the solid–liquid contact area of the super-hydrophobic coating with water droplets can be obtained. Additionally, the proportion of the gas–liquid contact area of the super-hydrophobic coating f_2_ is calculated as 87.51%. The results indicate that the actual contact area of the super-hydrophobic coating with water droplets only accounts for 12.49% of the total composite contact surface area, and the composite contact surface area occupied by air is estimated to be 87.51%. The calculation results indicate the excellent super-hydrophobic properties of the super-hydrophobic coating.

### 3.3. Chemical and Mechanical Durability

**Figure 3:**
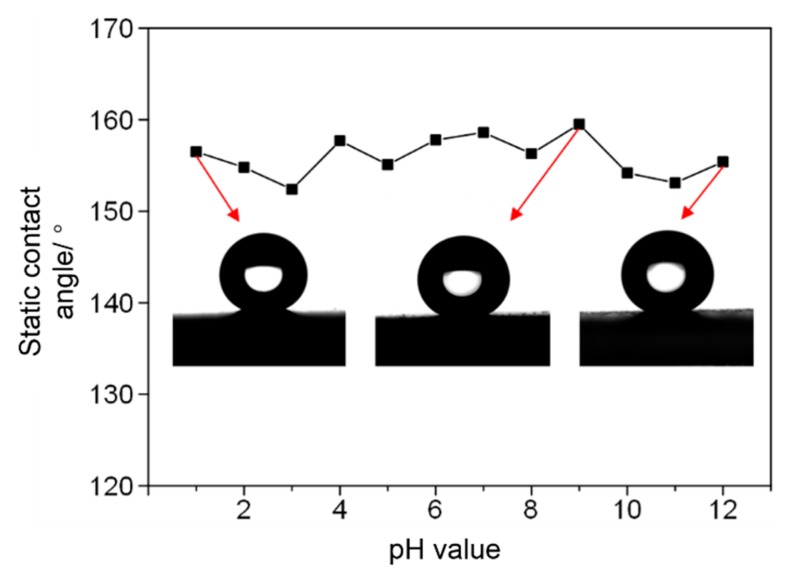
Static contact angle of super-hydrophobic methyl silicone resin/SiO_2_ composite coating after infiltrating with corrosive liquids with different pH values for 24 h.

The chemical durability of the super-hydrophobic methyl silicone resin/SiO_2_ composite coating was investigated. Figure 3 shows the static contact angle of the super-hydrophobic coating wetted by aqueous solutions with different pH values after 24 h. From the test results, after being wetted by corrosive liquids with pH values between 1 and 14, the static contact angle of super-hydrophobic surface is always greater than 150°. The minimum and maximum average static contact angle of the corrosive liquid are 152.4° and 159.5°, respectively.

**Figure 4:**
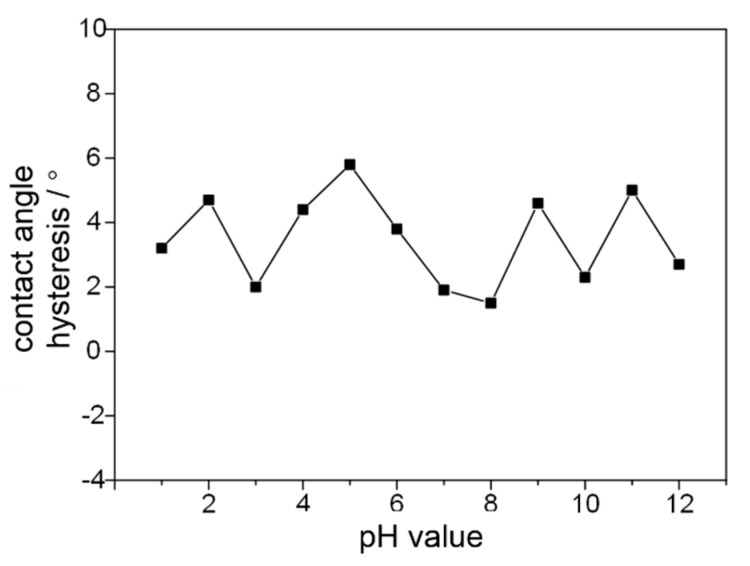
Water contact angle hysteresis of the super-hydrophobic methyl silicone resin/SiO_2_ composite coating after infiltrating with acidic and base liquids for 24 h.

Figure 4 shows the contact angle hysteresis of the super-hydrophobic coating wetted by corrosive liquids with pH values ranging from 1 to 14 after 24 h. The contact angle hysteresis of the water droplets stays low with a maximum average of 5.8° and a minimum average of 1.5°. Apparently, the corrosive liquids with different pH range have little influence on the static contact angle and contact angle hysteresis of the super-hydrophobic methyl silicone resin/SiO_2_ composite coating.

The tests above show that the methyl silicone resin/SiO_2_ composite coating maintains super-hydrophobic performance after the corrosion with acidic and alkaline solutions for 24 h, and demonstrates excellent acid and base tolerance. When acidic or basic corrosive liquids are dropped on the surface of super-hydrophobic coating, the OH-PDMS molecules and the methyl silicone molecules in the super-hydrophobic coatings come into direct contact with the corrosive liquids. The chemically stable OH-PDMS and methyl silicone molecules cannot be corroded by acidic or alkaline mediums. The great gas–liquid contact area of the super-hydrophobic coating reduces the contact area between the corrosive liquids and the super-hydrophobic methyl silicone resin/SiO_2_ composite surface. Both the chemical stability of the low-energy molecules and the low contact area with the corrosive liquids result in the corrosive durability of the super-hydrophobic methyl silicone resin/SiO_2_ composite coating. 

**Figure 5:**
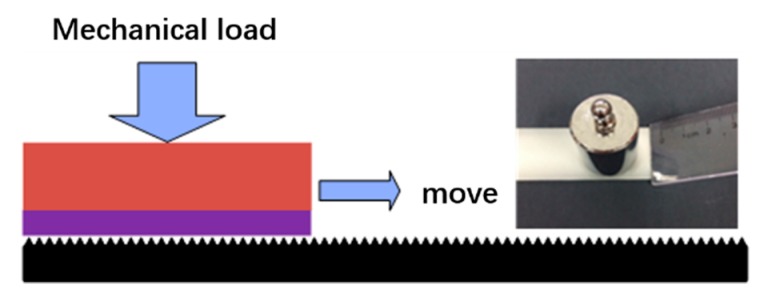
Schematic diagram of the wearing test of the super-hydrophobic surface.

**Figure 6:**
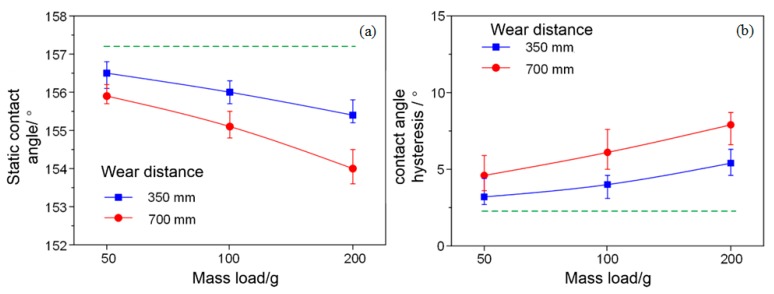
(**a**) Static water contact angles and (**b**) water contact angle hysteresis of the worn super-hydrophobic coatings.

The mechanical durability of the super-hydrophobic methyl silicone resin/SiO_2_ composite coating was investigated by sandpaper abrasion. The test process of sandpaper abrasion is shown in Figure 5. The super-hydrophobic coating was worn by a 1500 mesh sandpaper with different mechanical loads on it. After sandpaper abrasion of the super-hydrophobic methyl silicone resin/SiO_2_ composite coating at a speed of about 20 mm/s, the static contact angle and contact angle hysteresis of the worn super-hydrophobic coating were measured. Figure 6a shows the static water contact angle of the super-hydrophobic coatings under different mechanical loads (50 g, 100 g, and 200 g) and wearing distances (350 mm and 700 mm). For super-hydrophobic coatings with a wear distance of 350 mm, the static water contact angle decreases from 156.5° to 155.4° while the mechanical load increased from 50 g to 200 g. With the same wear distance, the static water contact angle of the super-hydrophobic coating gradually decreases with the greater mechanical load. For a super-hydrophobic coating with a mechanical load of 200 g, the static water contact angle decreases from 155.4° to 154.0° as the wear distance increases from 350 mm to 700 mm. With the same mechanical load, the static contact angle of the super-hydrophobic coating decreases as the wearing distance increases. Compared with the static water contact angle of the fresh super-hydrophobic coating (dashed line in the Figure 6a), the static water contact angle of the super-hydrophobic coating decreases slightly because of mechanical wearing, but remains over 150.0°. 

Figure 6b shows the contact angle hysteresis of the super-hydrophobic coating under different mechanical loads (50 g, 100 g, and 200 g) and wearing distances (350 mm and 700 mm). For the super-hydrophobic coating with the wear distance of 350 mm, the contact angle hysteresis increases from 3.2° to 5.4° when the mechanical load increases from 50 g to 200 g. The contact angle hysteresis of the super-hydrophobic coating gradually increases as the mechanical load increases in the case of the same wear distance. For the super-hydrophobic coating with a mechanical load of 200 g, the contact angle hysteresis increases from 5.4° to 7.9° when the wearing distance increases from 350 mm to 700 mm. The contact angle hysteresis of the super-hydrophobic coating also increases as the wearing distance increases for the same mechanical load. The contact angle hysteresis of the super-hydrophobic coating increases with mechanical wearing, but remains within 10° in the study, compared with the contact angle hysteresis of 2.3° for the unworn super-hydrophobic coating. The test results above show that the static water contact angle and the contact angle hysteresis of the super-hydrophobic coating degrades with the enhanced mechanical stress and wear distance. However, after severe mechanical tests, the methyl silicone resin/SiO_2_ composite coating stays super-hydrophobic.

**Figure 7:**
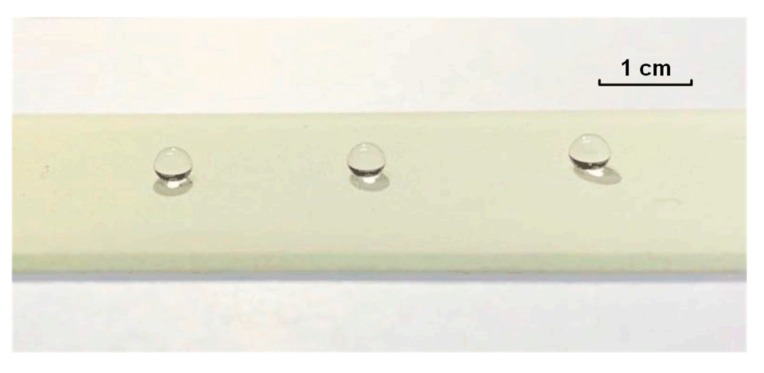
Hydrophobic performances of the super-hydrophobic coating after the sandpaper wearing test.

Figure 7 demonstrates a representative side view of water droplets on a super-hydrophobic coating after wearing for 100 mm with a 200 g mechanical load. The surface of the worn super-hydrophobic coating remains flat and free of visible scratches. The water droplets remain spherical in the worn area of the super-hydrophobic surface. In addition, water droplets easily slide off the slightly inclined super-hydrophobic surface. The results above indicate the great mechanical durability of the super-hydrophobic coating.

**Figure 8:**
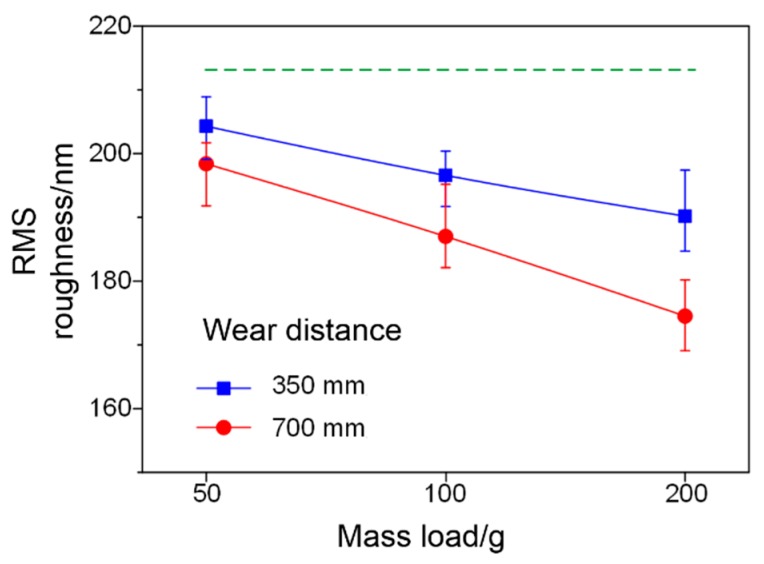
Root-mean-square (RMS) roughness of the worn super-hydrophobic coatings.

As shown in Figure 8, compared with the root-mean-square roughness of 213 nm for the fresh super-hydrophobic coating, the super-hydrophobic surface under the wear distance of 350 mm reduces the surface roughness from 204.3 nm to 190.2 nm with the mechanical load between 50 g and 200 g. For the super-hydrophobic coating with a mechanical load of 200 g, the surface root-mean-square roughness decreases from 190.2 nm to 174.5 nm as the wearing distance increases from 350 mm to 700 mm.

**Figure 9:**
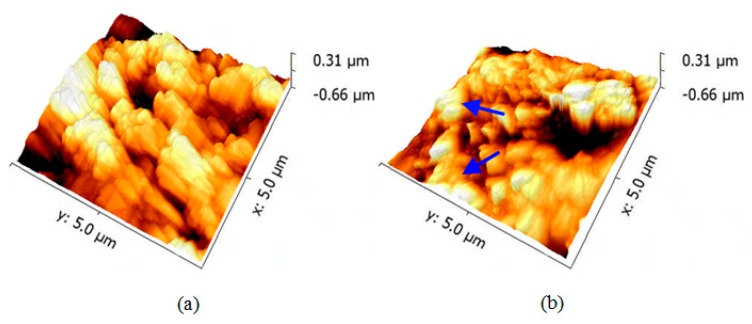
Atomic force microscope (AFM) images of the (**a**) as-prepared and (**b**) worn super-hydrophobic coatings.

Figure 9 shows the surface topography of the super-hydrophobic coating before and after mechanical abrasion for 700 mm with a mechanical load of 200 g. Micro-scaled scratches can be observed as shown in blue arrows on the rugged rough surface of the super-hydrophobic coating. Compared with the as-prepared super-hydrophobic surface, certain amounts of micro-protrusions at a size of around 1.2 μm disappeared on the worn super-hydrophobic surface. However, irregular micron-scale protrusions of around 0.4 μm with embedded nano-scale bumps are still visible on the surface of the super-hydrophobic coating.

For the high activity of the methyl silicone resin, the strong adhesion ability of the methyl silicone resin between the nano-SiO_2_ particles and the substrate renders the difficulty in the destruction of the rough structures on surface of the super-hydrophobic coating. The maintained binary micro/nano roughness enables the low contact ratio of the droplets with the rough surface, as well as the great air gap between the droplets and the coating. Thus, the worn methyl silicone resin/SiO_2_ composite surface demonstrates similar super-hydrophobic performances to the fresh one. 

### 3.4. Surface Adhesion and Hardness

**Figure 10:**
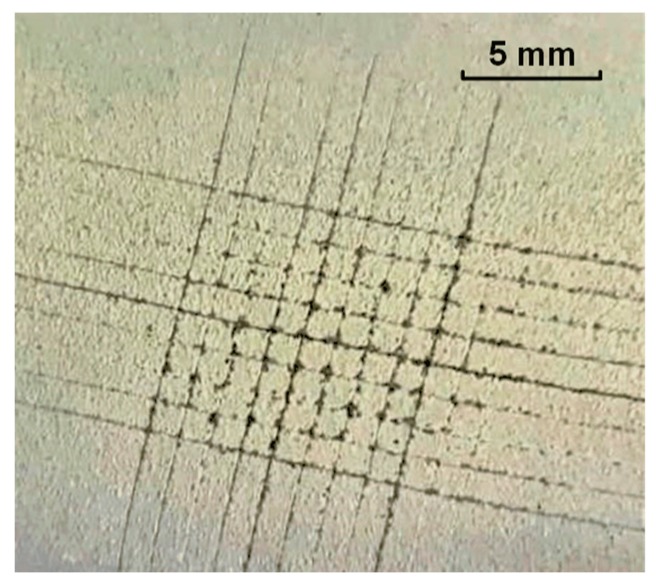
Surface adhesion of the super-hydrophobic surface by cross-cut tester.

Figure 10 shows the adhesion of the super-hydrophobic methyl silicone resin/SiO_2_ composite coating on the surface using the method of lattice grid cutting. The square edge has a few peeling marks, and most of the squares are intact without large-area blanks. According to the evaluation criteria of the adhesion of the coating [43], the adhesion of the methyl silicone resin/SiO_2_ composite coating with the glass substrate is determined to be grade 1. 

**Figure 11:**
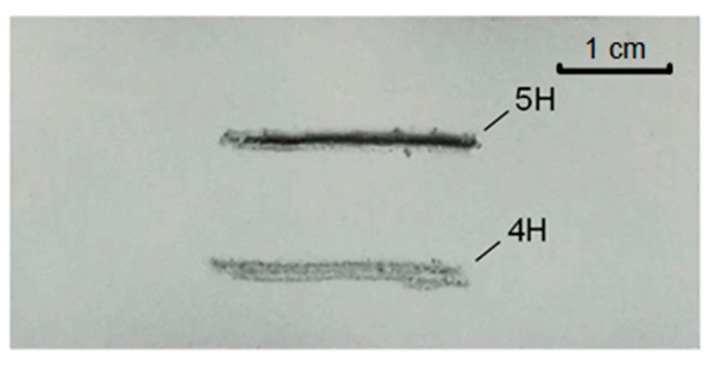
Surface hardness of the super-hydrophobic coating after the pencil test.

Figure 11 shows the surface hardness of the super-hydrophobic composite surface by pencil scratch with different hardness. After the pencil with a hardness of 4H crosses the surface of the super-hydrophobic coating, there is no damage caused on the coating surface. However, the surface of the super-hydrophobic coating gets scratched by the pencil with a hardness of 5H. On the basis of the standard of ASTM D3363 [44], the surface hardness of the super-hydrophobic methyl silicone resin/SiO_2_ composite coating is measured as 4H.

## 5. Conclusions

A durable super-hydrophobic coating on the surface of a glass slide is fabricated by air spraying of composites of methyl silicone resins and nano-sized silica (SiO_2_) particles. The as-prepared durable super-hydrophobic surface exhibits a water contact angle (WCA) of 157.2° and water contact angle hysteresis of 2.3°. SEM images indicate the existence of micro/nano hierarchical structures on super-hydrophobic surfaces. The adhesion strength and hardness of the surface is determined to be grade 1 and 4H, respectively. In the mechanical durability test, the micro/nano hierarchical structures remain on the surface of the super-hydrophobic coating after wearing for 700 mm with a mechanical load of 200 g. The rough surface retained after severe mechanical abrasion microscopically proves the durable origin of super-hydrophobic coating. The results demonstrate the feasibility of production of durable super-hydrophobic coating via enhancing its surface hardness and adhesion strength between the coating and the substrates.
